# Enhanced glutamate, IP3 and cAMP activity in the cerebral cortex of Unilateral 6-hydroxydopamine induced Parkinson's rats: Effect of 5-HT, GABA and bone marrow cell supplementation

**DOI:** 10.1186/1423-0127-18-5

**Published:** 2011-01-15

**Authors:** MS Nandhu, Jes Paul, Korah P Kuruvilla, Anitha Malat, Chinthu Romeo, CS Paulose

**Affiliations:** 1 Molecular Neurobiology and Cell Biology Unit, Centre for Neuroscience, Department of Biotechnology, Cochin University of Science and Technology, Cochin - 682 022, and Kerala, India

## Abstract

Parkinson's disease is characterized by progressive cell death in the substantia nigra pars compacta, which leads to dopamine depletion in the striatum and indirectly to cortical dysfunction. Increased glutamatergic transmission in the basal ganglia is implicated in the pathophysiology of Parkinson's disease and glutamate receptor mediated excitotoxicity has been suggested to be one of the possible causes of the neuronal degeneration. In the present study, the effects of serotonin, gamma-aminobutyric acid and bone marrow cells infused intranigrally to substantia nigra individually and in combination on unilateral 6-hydroxydopamine induced Parkinson's rat model was analyzed. Scatchard analysis of total glutamate and NMDA receptor binding parameters showed a significant increase in B_max _(P < 0.001) in the cerebral cortex of 6-hydroxydopamine infused rat compared to control. Real Time PCR amplification of NMDA2B, mGluR5, bax, and ubiquitin carboxy-terminal hydrolase were up regulated in cerebral cortex of 6-hydroxydopamine infused rats compared to control. Gene expression studies of GLAST, ά-Synuclien and Cyclic AMP response element-binding protein showed a significant (P < 0.001) down regulation in 6-OHDA infused rats compared to control. Behavioural studies were carried out to confirm the biochemical and molecular studies. Serotonin and GABA along with bone marrow cells in combination showed reversal of glutamate receptors and behaviour abnormality shown in the Parkinson's rat model. The therapeutic significance in Parkinson's disease is of prominence.

## Background

Parkinson's disease (PD), one of the most prevalent neurodegenerative disorders among the elderly population, is charecterised by dopamine neurons degeneration in the substantia nigra pars compacta. Which makes an impact on ascending adrenergic and serotonergic networks, frontocortical cholinergic projections, and a diversity of neuronal circuits located not only in the brain (from the cortex to the medulla), but even in the spinal cord and sympathetic nervous system [1,2]. Recent studies have shown abnormal mitochondrial content and function, also an increased oxidative stress and oxidative responses in the cerebral cortex in PD [3]. According to the classical model of basal ganglia organization, the usual facilitating effect of thalamic projections to the cerebral cortex is reduced in PD [4]. The motor dysfunction of PD is generally accompanied by depressed affect and cognitive impairment, comprising the triad of deficits that most profoundly interfere with patient quality of life [5]. Antagonising excitotoxicity has been considered to have therapeutic potential for the treatment of PD. Glutamate neurotransmission plays an integral role in basal ganglia functioning especially in the striatum, where the balance of glutamate and dopamine is critical but also in the substantia nigra which receives glutamatergic input from the subthalamic nucleus and cortex [6]. At physiological concentrations, glutamate mediates learning and memory processes [7]. However, at high concentrations, glutamate acts as a neurotoxin and promotes neuronal cell injury and death in PD [8].

Cell transplantation to replace lost neurons is a promising approach for the treatment of progressive neurodegenerative diseases. Autologous bone marrow cells (BMC) can be used as a source of progenitor cells for the central nervous system. It circumvents both ethical and immunological constraints related with stem cell therapy. Neurotransmitter's combination as therapeutic agents for cell proliferation and differentiation is a novel approach. In rats, 5-HT neurons in the brainstem raphe are among the first neurons to differentiate in the brain and play a key role in regulating neurogenesis [9]. Lauder and Krebs [10] reported that parachlorophenylalanine (PCPA), a 5-HT synthesis inhibitor, retarded neuronal maturation, while mild stress, a releaser of hormones, accelerated neuronal differentiation. These workers defined differentiation as the cessation of cell division measured by incorporation of ^3^H-thymidine. Since then, many other workers have shown a role for serotonin in neuronal differentiation [11]. GABA, the main inhibitory neurotransmitter in the mature CNS, was recently implicated in playing a complex role during neurogenesis [12,13]. Through embryonic development, GABA was demonstrated as acting as a chemo-attractant and being involved in the regulation of progenitor cell proliferation. For example, GABA induces migration and motility of acutely dissociated embryonic cortical neurons [12,14]. GABA acts as a trophic factor not solely during prenatal neurogenesis but also in the postnatal period in injured tissue. The effect of GABA involves stimulation of cell proliferation and Nerve growth factor secretion [15]. We have previously shown that Serotonin (5HT) and Gamma aminobutyric acid (GABA) acting through specific receptor subtypes 5HT_2 _[16] and GABA_A _[17] respectively, control cell proliferation and act as comitogens. Our present study demonstrates the structural and molecular changes of 6-OHDA infused unilateral Parkinson's model using 5-HT, GABA and BMC individually and in combination.

## Materials and methods

### Animals

Experiments were carried out on adult male Wistar rats of 250-300 g body weight purchased from Kerala Agricultural University, Mannuthy, were used for all experiments. They were housed in separate cages under 12 hrs light and 12 hrs dark periods and were maintained on standard food pellets and water *ad libitum*. All animal care and procedures were taken in accordance with the Institutional, National Institute of Health guidelines and CPCSEA guidelines.

### Chemicals used and their sources

Biochemicals, Tri-reagent kit, primary and secondary antibodies used in the present study were purchased from Sigma Chemical Co., St. Louis, USA. All other reagents were of analytical grade purchased locally. L-[G-^3^H]Glutamic acid (Sp. Activity 49.0 Ci/mmol) was purchased from Amersham Life Science, UK. (+)-[3-^3^H] MK-801 (Sp. Activity 27.5 Ci/mmol) was purchased from Perkin Elmer, Boston, MA, USA. ABI PRISM High Capacity cDNA Archive kit, Primers and Taqman probes for Real-Time PCR were purchased from Applied Biosystems, Foster City, CA, USA.

### Experimental design

The experimental rats were divided into the following groups i) Control ii) 6-OHDA infused (6-OHDA) iii) 6-OHDA infused supplemented with Serotonin (6-OHDA + 5-HT) and iv) 6-OHDA infused supplemented with GABA (6-OHDA + GABA) v) 6-OHDA infused supplemented with Bone marrow cells (isolated from rats on femur) (6-OHDA + BMC) vi) 6-OHDA infused supplemented with 5-HT and BMC (6-OHDA+5-HT+BMC) vii) 6-OHDA infused supplemented with GABA and BMC (6-OHDA+ GABA+BMC) viii) 6-OHDA infused supplemented with 5-HT, GABA and BMC (6-OHDA+5-HT+GABA+BMC). Each group consisted of 6-8 animals.

Rats were anesthetized with Chloryl Hydrate (450 mg/kg body weight. i.p.). The animal was placed in the flat skull position on a cotton bed on a stereotaxic frame (Benchmark™, USA) with incisor bar fixed at 3.5 mm below the interaural line. 6-OHDA (8 μg in 1 μl in 0.2% ascorbic acid) was infused into the right Substantia nigra Pars compacta (SNpc) at a flow rate of 0.2 μl/min. After stopping the infusion of the toxin, the probe was kept in the same position for a further 5 min for complete diffusion of the drug and then slowly retracted. All the groups except Control group were infused with 6-OHDA and in control animals, 1 μl of the vehicle (0.2% ascorbic acid) was infused into the right SNpc.

### Rotational behavior

Amphetamine-induced rotational behavior was assessed as described earlier [18]. Rats were tested with amphetamine on the 14^th ^day after intranigral injection of 6-OHDA and with apomorphine on the 16^th ^day. Animals that had completed a 360^◦ ^circle towards the intact (contralateral) and the lesioned (ipsilateral) sides were counted for 60 min continuously and recorded separately (animals that showed no significant contralateral rotations were excluded from the study).

### Treatment

**Figure 1:**
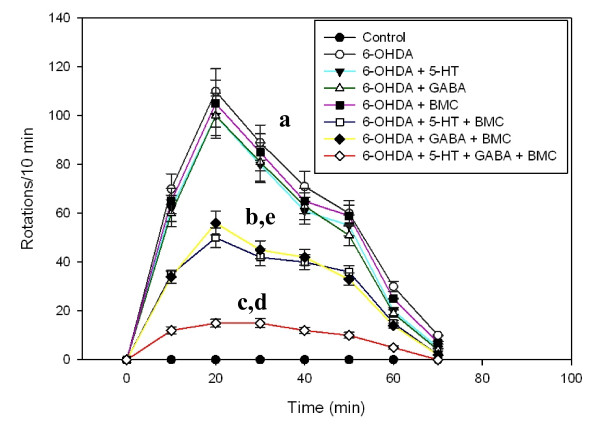
**Apomorphine induced rotational behaviour in experimental rats**. Adult male Wistar rats were intranigrally infused with 6-OHDA (8 μg in 1 μl). Bone marrow cell and neurotransmitters were infused individually and in combination in denervated striatum on the 18^th ^day. Animals were injected with apomorphine (1 mg/kg; s.c.) on the 30^th ^day and the apomorphine-induced rotations were recorded for every 10 min duration for a period of 70 min. Values are Mean ± S.E.M of 4-6 separate experiments. Each group consist 6-8 rats. ^a ^p < 0.001, ^b ^p < 0.01, ^c ^P < 0.05 when compared to Control. ^d ^p < 0.001, ^e ^p < 0.01 when compared to 6-OHDA group.

On the 18^th ^day and Stereotaxic single dose of 1 μl of 5-HT (10 μg/μl), GABA(10 μg/μl) and 10 μl of Bone marrow cell (BMC) (10^6 ^Cells/ml) suspension individually and in combination (combinational treatment) was infused into the right SNpc at a flow rate of 0.2 μl/min into the respective groups. On the 30^th ^day and the apomorphine-induced rotations were recorded for every 10 min duration for a period of 70 min (Figure 1). All the control and experimental rats were sacrificed by decapitation. The cerebral cortex was dissected out quickly over ice [19] and the tissues were stored at -80°C for various experiments.

### Quantification Dopamine in the cerebral cortex

The monoamines were assayed according to the modified procedure of Paulose et al., [20]. The cerebral cortex of experimental gropes of rats was homogenised in 0.4N perchloric acid. The homogenate was then centrifuged at 5000 × g for 10 minutes at 4°C in a Sigma 3K30 refrigerated centrifuge and the clear supernatant was filtered through 0.22 μm HPLC grade filters and used for HPLC analysis.

Dopamine (DA) contents was determined in high performance liquid chromatography (HPLC) with electrochemical detector (ECD) (Waters, USA) fitted with CLC-ODS reverse phase column of 5 μm particle size. The mobile phase consisted of 50 mM sodium phosphate dibasic, 0.03M citric acid, 0.1 mM EDTA, 0.6 mM sodium octyl sulfonate, 15% methanol. The pH was adjusted to 3.25 with orthophosphoric acid, filtered through 0.22 μm filter (Millipore) and degassed. A Waters (model 515, Milford, USA) pump was used to deliver the solvent at a rate of 1 ml/minute. The neurotransmitters and their metabolites were identified by amperometric detection using an electrochemical detector (Waters, model 2465) with a reduction potential of +0.80 V. Twenty microlitre aliquots of the acidified supernatant were injected into the system for detection. The peaks were identified by relative retention times compared with external standards and quantitatively estimated using an integrator (Empower software) interfaced with the detector.

### Glutamate content analysis in the cerebral cortex

Glutamate contents in the cerebral cortex of experimental groups were quantified by displacement method using modified procedure of Enna and Snyder [21]. Tissue was homogenized in 20 volumes of 0.32 M sucrose, 10 mM Tris/HCl and 1 mM MgCl_2 _buffer, pH 7.4, with a polytron homogenizer. The homogenate was centrifuged twice at 27,000 × g for 15 minutes. The supernatant were pooled and used for the assay. The incubation mixture for glutamate quantification contained 1 nM [^3^H] glutamate with and without glutamate at a concentration range of 10^-9 ^M to 10^-4 ^M.

### Glutamate Receptor Binding Studies Using [^3^H]Glutamate

Membranes were prepared according to the modified method of Timothy *et al*., [22]. Membranes were incubated in 0.25 ml reaction mixture containing 25 mM Tris-HCl, pH 7.4, 5 mM MgCl_2 _and 20 nM to 350 nM of [^3^H]Glutamate containing 0.2 mg to 0.3 mg protein concentrations. Nonspecific binding was determined by adding 350 μM nonradioactive glutamate to the reaction mixture in a parallel assay.

### NMDA Receptor Binding Studies Using [^3^H] MK-801

The membrane fractions were prepared by a modification of the method described by Hoffman *et al*., [23]. The [^3^H] MK-801 binding saturation assay was performed in a concentration range of 0.25 to 50 nM at 23°C in an assay medium containing 10 mM HEPES, pH 7.0, 200 - 250 μg of protein, 100 μM glycine and 100 μM glutamate. Specific [^3^H] MK-801 binding was obtained by subtracting nonspecific binding in the presence of 100 μM unlabeled MK-801 from the total binding.

### Protein Determination

Protein was measured [24] using bovine serum albumin as standard. The intensity of the purple blue colour formed was proportional to the amount of protein which was read in a spectrophotometer at 660 nm.

### Analysis of the Receptor-Binding Data

#### Linear Regression Analysis for Scatchard Plots

The data were analysed [25]. The specific binding was determined by subtracting non-specific binding from the total. The binding parameters, maximal binding (*B_max_*) and equilibrium dissociation constant (*K_d_*), were derived by linear regression analysis by plotting the specific binding of the radioligand on the *X*-axis and bound/free on the *Y*-axis. The maximal binding is a measure of the total number of receptors present in the tissue and the equilibrium dissociation constant is the measure of the affinity of the receptors for the radioligand. The *K_d _*is inversely related to receptor affinity.

#### Quantification of IP3

The cerebral cortex was homogenised in a polytron homogeniser in 50 mM Tris-HCl buffer, pH.7.4, containing 1 mM EDTA to obtain a 15% homogenate. The homogenate was then centrifuged at 40,000 × g for 15 min. and the supernatant was transferred to fresh tubes for IP3 assay using [^3^H]IP3 Biotrak Assay System kit. The unknown concentrations were determined from the standard curve using appropriate dilutions and calculated for picomoles/g wt. of the tissue.

A standard curve was plotted with %B/Bo on the Y-axis and IP3 concentration (pmoles/tube) on the X-axis of a semi-log graph paper. %B/B_o _was calculated as:

$$\frac{\left(\text{Standard or sample cpm }-\text{ NSB cpm}\right)}{\left({\text{B}}_{0}\text{cpm }-\text{ NSB cpm}\right)}\;\times \text{ 100}$$

NSB- non specific binding and B_0 _- zero binding. IP3 concentrations in the samples were determined by interpolation from the plotted standard curve.

#### cAMP content in the cerebral cortex of control and experimental rats

The cerebral cortex was homogenised in a polytron homogeniser with cold 50 mM Tris-HCl buffer, pH 7.4, containing 1 mM EDTA to obtain a 15% homogenate. The homogenate was then centrifuged at 40,000 × g for 15 min and the supernatant was transferred to fresh tubes for cAMP assay using [^3^H]cAMP Biotrak Assay System kit. The unknown concentrations were determined from the standard curve using appropriate dilutions and calculated for picomoles/g wt. of the tissue.

C_o_/C_x _was plotted on the Y-axis against picomoles of inactive cAMP on the X- axis of a linear graph paper, where C_o _is the counts per minute bound in the absence of unlabelled cAMP and C_x _was the counts per minute bound in the presence of standard or unknown unlabelled cAMP. From the C_o_/C_x _value for the sample, the number of picomoles of unknown cAMP was calculated.

#### Analysis of gene expression by real-time polymerase chain reaction

RNA was isolated using Tri reagent. Total cDNA synthesis was performed using ABI PRISM cDNA Archive kit. Real-Time PCR assays were performed in 96-well plates in ABI 7300 Real-Time PCR instrument (Applied Biosystems). PCR analyses were conducted with gene-specific primers and fluorescently labelled Taqman probe of NMDA2B, mGluR5, GLAST, bax, ά-Synuclien, ubiquitin carboxy-terminal hydrolase and Cyclic AMP response element-binding protein (CREB) (designed by Applied Biosystems). Endogenous control, β-actin, was labeled with a report dye, VIC.

#### NMDA2B and mGluR5 Receptor Expression using Confocal Microscope

The rat was transcardially perfused with PBS, pH- 7.4, followed by 4% paraformaldehyde in PBS [26]. 10 μm brain sections were cut using Cryostat (Leica, CM1510 S). Brain slices were incubated overnight at 4°C with rat primary antibody for NMDA2B and mGluR5. After overnight incubation brain slices were incubated with the secondary antibody of FITC. The sections were observed and photographed using confocal imaging system (Leica SP 5).

#### Statistical Analysis

Statistical evaluations were done with analysis of variance (ANOVA), using GraphPad Instat (version 2.04a, San Diego, USA). Student Newman-Keuls test was used to compare different groups after ANOVA. Linear regression Scatchard plots were made using SIGMA PLOT (Ver 2.03). Relative Quantification Software was used for analyzing Real-Time PCR results.

## Results

### Dopamine content in the cerebral cortex

6-OHDA infusion in to the SNpc resulted in a significant (p < 0.001) decrease in dopamine content in the cerebral cortex of PD rats. Dopamine production was lower in the rats treated with 5-HT, GABA, BMC individually. Combinational treatment significantly reversed the dopamine content to near control level (Table 1).

**Table 1:** Dopamine Content (pmol/mg protein) in the Cerebral cortex of experimental rats

Animal status	Dopamine Content (pmol/mg protein)
Control	57.05 ± 2.90

6-OHDA	3.57 ± 1.34 ^a^

6-OHDA +5HT	14.21 ± 1.51 ^b,f^

6-OHDA +GABA	13.38 ± 1.64 ^b,f^

6-OHDA +BMC	5.24 ± 2.25 ^a^

6-OHDA +5HT + BMC	37.82 ± 3.47^c,e^

6-OHDA + GABA + BMC	39.63 ± 3.82 ^c,e^

6-OHDA +5HT + GABA+ BMC	50.41 ± 3.02 ^c,d^

Values are Mean ± S.E.M of 4-6 separate experiments. Each group consist 6-8 rats.

^a ^p < 0.001, ^b ^p < 0.01, ^c ^P < 0.05 when compared to Control

^d ^p < 0.001, ^e ^p < 0.01, ^f ^P < 0.05 when compared to 6-OHDA group.

C - Control, 6-OHDA - 6-OHDA infused, 6-OHDA +5-HT - 6-OHDA infused treated with Serotonin, 6-OHDA +GABA - 6-OHDA infused treated with GABA, 6-OHDA +BMC- 6-OHDA infused treated with BMC, 6-OHDA +5-HT+BMC- 6-OHDA infused treated with Serotonin and BMC, 6-OHDA + GABA +BMC- 6-OHDA infused treated with GABA and BMC, 6-OHDA +5-HT + GABA+ BMC- 6-OHDA infused treated with Serotonin, GABA and BMC.

### Glutamate, IP3 and cAMP content in the cerebral cortex

**Figure 2:**
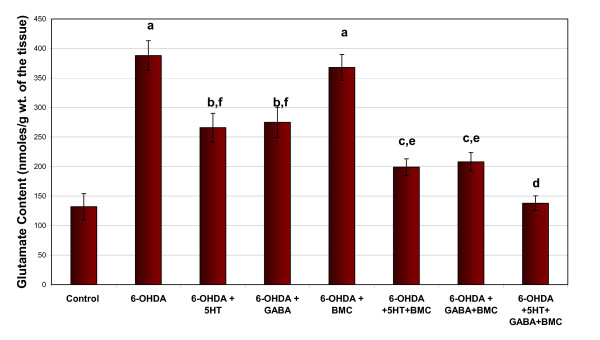
**Representative graph showing Glutamate content in the cerebral cortex of experimental rats**. Values are Mean ± S.E.M. of 4-6 separate experiments. Each group consists of 6-8 rats. ^a ^p < 0.001, ^b ^p < 0.01, ^c ^P < 0.05 when compared to Control, ^d ^p < 0.001, ^e ^p < 0.01, ^f ^P < 0.05 when compared to 6-OHDA group. C - Control, 6-OHDA - 6-OHDA infused, 6-OHDA +5-HT - 6-OHDA infused treated with Serotonin, 6-OHDA +GABA - 6-OHDA infused treated with GABA, 6-OHDA +BMC- 6-OHDA infused treated with BMC, 6-OHDA +5-HT+BMC- 6-OHDA infused treated with Serotonin and BMC, 6-OHDA + GABA +BMC- 6-OHDA infused treated with GABA and BMC, 6-OHDA +5-HT + GABA+ BMC- 6-OHDA infused treated with Serotonin, GABA and BMC.

**Figure 3:**
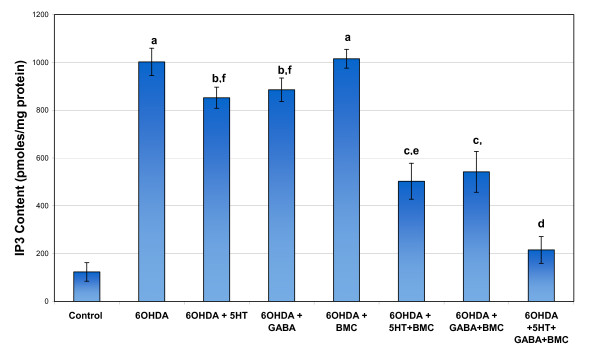
**Representative graph showing IP3 content in the cerebral cortex of experimental rats**. Values are Mean ± S.E.M. of 4-6 separate experiments. Each group consists of 6-8 rats. ^a ^p < 0.001, ^b ^p < 0.01, ^c ^P < 0.05 when compared to Control, ^d ^p < 0.001, ^e ^p < 0.01, ^f ^P < 0.05 when compared to 6-OHDA group. C - Control, 6-OHDA - 6-OHDA infused, 6-OHDA +5-HT - 6-OHDA infused treated with Serotonin, 6-OHDA +GABA - 6-OHDA infused treated with GABA, 6-OHDA +BMC- 6-OHDA infused treated with BMC, 6-OHDA +5-HT+BMC- 6-OHDA infused treated with Serotonin and BMC, 6-OHDA + GABA +BMC- 6-OHDA infused treated with GABA and BMC, 6-OHDA +5-HT + GABA+ BMC- 6-OHDA infused treated with Serotonin, GABA and BMC.

**Figure 4:**
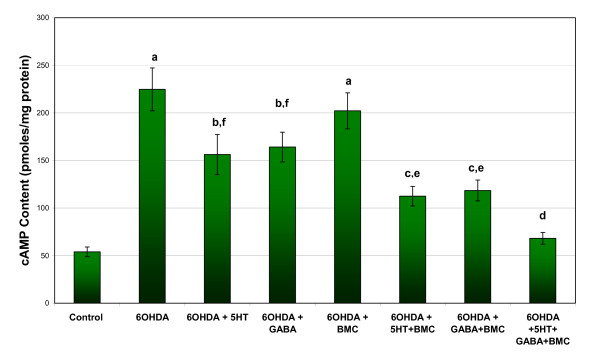
**Representative graph showing cAMP content in the cerebral cortex of experimental rats**. Values are Mean ± S.E.M. of 4-6 separate experiments. Each group consists of 6-8 rats. ^a ^p < 0.001, ^b ^p < 0.01, ^c ^P < 0.05 when compared to Control, ^d ^p < 0.001, ^e ^p < 0.01, ^f ^P < 0.05 when compared to 6-OHDA group. C - Control, 6-OHDA - 6-OHDA infused, 6-OHDA +5-HT - 6-OHDA infused treated with Serotonin, 6-OHDA +GABA - 6-OHDA infused treated with GABA, 6-OHDA +BMC- 6-OHDA infused treated with BMC, 6-OHDA +5-HT+BMC- 6-OHDA infused treated with Serotonin and BMC, 6-OHDA + GABA +BMC- 6-OHDA infused treated with GABA and BMC, 6-OHDA +5-HT + GABA+ BMC- 6-OHDA infused treated with Serotonin, GABA and BMC.

Glutamate, IP3 and cAMP content showed a significant increase in cerebral cortex of 6-OHDA rats compared to control. Individual treatment with BMC, 5-HT and GABA didn't alter the changes. Combinational treatment significantly reversed the content values to near control level (Figure 2, 3 and 4).

### Total glutamate receptor analysis

Scatchard analysis of [^3^H]glutamate against glutamate in cerebral cortex of 6-OHDA group of rats showed a significant (p < 0.001) increase in B_max _compared to control rats. 6-OHDA+BMC group treated rats didn't reverse these changes. Combinational treatment significantly (p < 0.001) reversed these changes to near control. There was no significant change in K_d _in all experimental groups (Table 2).

**Table 2:** Scatchard Analysis of Glutamate receptors using [^3^H]Glutamate binding against glutamate in the Cerebral cortex of experimental rats

Animal Status	B_max _(fmoles/mg protein)	K_d _(nM)
Control	1584.04 ± 14.12	146.39 ± 16.41

6-OHDA	3598.40 ± 35.88^a^	138.58 ± 17.12

6-OHDA +5HT	1892.12 ± 18.41^b,f^	131.24 ± 19.85

6-OHDA +GABA	1984.05 ± 24.25^b,f^	128.12 ± 18.24

6-OHDA +BMC	3295.12 ± 29.12^a, f^	145.15 ± 11.22

6-OHDA +5HT + BMC	1775.41 ± 13.65^b,e^	125.13 ± 18.14

6-OHDA + GABA + BMC	1776.11 ± 14.21^b,e^	124.22 ± 22.11

6-OHDA +5HT + GABA+ BMC	1711.51 ± 10.18^c,d^	155.23 ± 15.26

Values are Mean ± S.E.M of 4-6 separate experiments. Each group consist 6-8 rats.

B_max _- Maximal binding; K_d _- Dissociation constant

^a ^p < 0.001, ^b ^p < 0.01, ^c ^P < 0.05 when compared to Control

^d ^p < 0.001, ^e ^p < 0.01, ^f ^P < 0.05 when compared to 6-OHDA group.

C - Control, 6-OHDA - 6-OHDA infused, 6-OHDA +5-HT - 6-OHDA infused treated with Serotonin, 6-OHDA +GABA - 6-OHDA infused treated with GABA, 6-OHDA +BMC- 6-OHDA infused treated with BMC, 6-OHDA +5-HT+BMC- 6-OHDA infused treated with Serotonin and BMC, 6-OHDA + GABA +BMC- 6-OHDA infused treated with GABA and BMC, 6-OHDA +5-HT + GABA+ BMC- 6-OHDA infused treated with Serotonin, GABA and BMC.

### NMDA receptor analysis

Scatchard analysis of [^3^H]MK-801 against MK-801 in cerebral cortex of 6-OHDA group of rats showed a significant (p < 0.001) up regulation in B_max _compared to control rats. Individual treatment group rats didn't reverse these changes. Combinational treatment significantly (p < 0.001) reversed these changes to near control. There was no significant change in K_d _in all experimental groups indicating that there is no change in the affinity of the receptors. This increased B_max _reflected an increased number of NMDA receptors in the experimental groups (Table 3).

**Table 3:** Scatchard Analysis of NMDA receptor using [^3^H] MK-801 binding against MK-801 in the Cerebral cortex of Control, 6-OHDA infused, 6-OHDA+5HT, 6-OHDA+GABA and 6-OHDA+BMC treated rats

Animal Status	B_max _(fmoles/mg protein)	K_d _(nM)
Control	261.60 ± 11.05	0.63 ± 0.11

6-OHDA	754.88 ± 16.28^a^	0.82 ± 0.18

6-OHDA + 5HT	619.28 ± 19.95^b,f^	0.75 ± 0.12

6-OHDA + GABA	638.24 ± 20.48^b,f^	0.77 ± 0.10

6-OHDA + BMC	669.92 ± 11.71^a,f^	0.80 ± 0.09

6-OHDA +5HT + BMC	328.33 ± 26.87^c,e^	0.72 ± 0.14

6-OHDA + GABA + BMC	344.96 ± 24.12^c,e^	0.65 ± 0.10

6-OHDA +5HT + GABA+ BMC	274.04 ± 15.12^c,d^	0.74 ± 0.08

Values are Mean ± S.E.M of 4-6 separate experiments. Each group consist 6-8 rats.

B_max _- Maximal binding; K_d _- Dissociation constant

^a ^p < 0.001, ^b ^p < 0.01, ^c ^P < 0.05 when compared to Control,

^d ^p < 0.001, ^e ^p < 0.01, ^f ^P < 0.05 when compared to 6-OHDA group.

C - Control, 6-OHDA - 6-OHDA infused, 6-OHDA +5-HT - 6-OHDA infused treated with Serotonin, 6-OHDA +GABA - 6-OHDA infused treated with GABA, 6-OHDA +BMC- 6-OHDA infused treated with BMC, 6-OHDA +5-HT+BMC- 6-OHDA infused treated with Serotonin and BMC, 6-OHDA + GABA +BMC- 6-OHDA infused treated with GABA and BMC, 6-OHDA +5-HT + GABA+ BMC- 6-OHDA infused treated with Serotonin, GABA and BMC.

### Real time PCR analysis of NMDA2B, mGluR5, GLAST, bax, ά-Synuclien, ubiquitin carboxy-terminal hydrolase and CREB

Gene expression studies of NMDA2B, mGluR5, bax and ubiquitin carboxy-terminal hydrolase showed a significant (P < 0.001) up regulation in 6-OHDA infused rats compared to control. At the same time the expression of the GLAST, ά-Synuclien and CREB showed a significant (P < 0.001) down regulation in 6-OHDA infused rats compared to control. Combinational treatment significantly reversed these changes back to near control (Table 4, 5).

**Table 4:** Real Time PCR amplification of mGluR5, NMDA2B and GLAST mRNA in the Cerebral cortex of experimental rats

Animal Status	Log RQ		
	
	mGluR5	NMDA2B	GLAST
Control	0	0	0

6-OHDA	3.55 ± 0.24^a^	2.14 ± 0.12^a^	-2.03 ± 0.11^a^

6-OHDA +5HT	2.56 ± 0.12^b, f^	1.65 ± 0.22^b, f^	-1.71 ± 0.14^b, f^

6-OHDA +GABA	2.64 ± 0.22^b, f^	1.68 ± 0.19^b, f^	-1.81 ± 0.08^b,f^

6-OHDA +BMC	3.41 ± 0.24^a^	2.10 ± 0.18^a^	-2.00 ± 0.06^a^

6-OHDA +5HT + BMC	1.52 ± 0.29^c, e^	0.89 ± 0.15^c, e^	-1.11 ± 0.19^c, e^

6-OHDA + GABA + BMC	1.84 ± 0.19^c, e^	0.92 ± 0.18^c, e^	-1.13 ± 0.12^c, e^

6-OHDA +5HT + GABA+ BMC	0.81 ± 0.10^d^	0.41 ± 0.12^d^	-0.32 ± 0.12^d^

Values are Mean ± S.E.M of 4-6 separate experiments. Each group consist 6-8 rats.

^a ^p < 0.001, ^b ^p < 0.01, ^c ^P < 0.05 when compared to Control,

^d ^p < 0.001, ^e ^p < 0.01, ^f ^P < 0.05 when compared to 6-OHDA group.

C - Control, 6-OHDA - 6-OHDA infused, 6-OHDA +5-HT - 6-OHDA infused treated with Serotonin, 6-OHDA +GABA - 6-OHDA infused treated with GABA, 6-OHDA +BMC- 6-OHDA infused treated with BMC, 6-OHDA +5-HT+BMC- 6-OHDA infused treated with Serotonin and BMC, 6-OHDA + GABA +BMC- 6-OHDA infused treated with GABA and BMC, 6-OHDA +5-HT + GABA+ BMC- 6-OHDA infused treated with Serotonin, GABA and BMC. The relative ratios of mRNA levels were calculated using the ΔΔCT method normalized with β-actin CT value as the internal control and Control CT-value as the calibrator.

**Table 5:** Real Time PCR amplification of bax, ubiquitin carboxy-terminal hydrolase, α-Synuclien and CREB mRNA in the Cerebral cortex of experimental rats

Animal Status	Log RQ			
	
	bax	ubiquitin carboxy-terminal hydrolase	α-Synuclien	CREB
Control	0	0	0	0

6-OHDA	1.96 ± 0.18^a^	0.99 ± 0.06 ^a^	-3.12 ± 0.31^a^	-2.91 ± 0.22^a^

6-OHDA +5HT	1.02 ± 0.19 ^b, f^	0.51 ± 0.05 ^b, f^	-1.41 ± 0.29^b,e^	-1.32 ± 0.13^b, f^

6-OHDA +GABA	1.06 ± 0.11 ^b, f^	0.50 ± 0.07 ^b, f^	-1.55 ± 0.26^b,e^	-1.43 ± 0.12^b, f^

6-OHDA +BMC	1.79 ± 0.10^a^	0.98 ± 0.04 ^a^	-2.99 ± 0.24^a^	-2.65 ± 0.21^a^

6-OHDA +5HT + BMC	0.64 ± 0.10 ^c, e^	0.23 ± 0.06^c, e^	0.12 ± 0.09^d^	-0.56 ± 0.08^c, e^

6-OHDA + GABA + BMC	0.61 ± 0.07^c,e^	0.26 ± 0.04^c, e^	0.13 ± 0.12^d^	-0.59 ± 0.09^c, e^

6-OHDA +5HT + GABA+ BMC	0.29 ± 0.06^d^	0.11 ± 0.02^d^	0.41 ± 0.13^d^	0.09 ± 0.03^d^

Values are Mean ± S.E.M of 4-6 separate experiments. Each group consist 6-8 rats.

^a ^p < 0.001, ^b ^p < 0.01, ^c ^P < 0.05 when compared to Control,

^d ^p < 0.001, ^e ^p < 0.01, ^f ^P < 0.05 when compared to 6-OHDA group.

C - Control, 6-OHDA - 6-OHDA infused, 6-OHDA +5-HT - 6-OHDA infused treated with Serotonin, 6-OHDA +GABA - 6-OHDA infused treated with GABA, 6-OHDA +BMC- 6-OHDA infused treated with BMC, 6-OHDA +5-HT+BMC- 6-OHDA infused treated with Serotonin and BMC, 6-OHDA + GABA +BMC- 6-OHDA infused treated with GABA and BMC, 6-OHDA +5-HT + GABA+ BMC- 6-OHDA infused treated with Serotonin, GABA and BMC. The relative ratios of mRNA levels were calculated using the ΔΔCT method normalized with β-actin CT value as the internal control and Control CT-value as the calibrator.

### Immunohistochemistry of mGLUR5 and NMDAR1 receptor antibody staining

**Figure 5:**
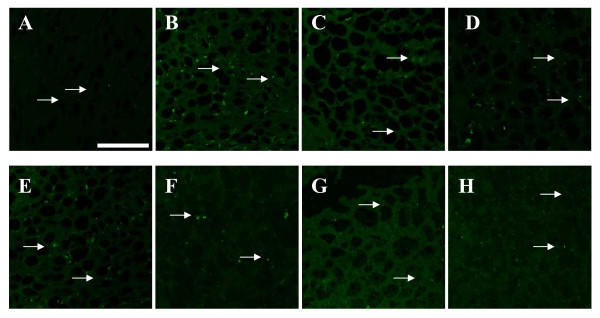
**mGluR5 receptors expression in the cerebral cortex of experimental rats using immunofluorescent mGluR5 receptor specific primary antibody and FITC as secondary antibody**. a - Control, b - 6-OHDA infused, c- 6-OHDA infused treated with Serotonin, d - 6-OHDA infused treated with GABA, e- 6-OHDA infused treated with BMC, f- 6-OHDA infused treated with Serotonin and BMC, g- 6-OHDA infused treated with GABA and BMC, h- 6-OHDA infused treated with Serotonin, GABA and BMC. There was an up regulation of mGluR5 receptors in the cerebral cortex of experimental rats when compared to control rats. The scale bars represent 75 μm.

**Figure 6:**
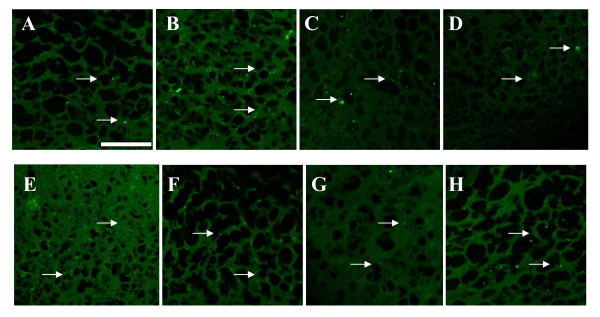
**NMDA2B receptors expression in the cerebral cortex of experimental rats using immunofluorescent NMDA2B receptor specific primary antibody and FITC as secondary antibody**. a - Control, b - 6-OHDA infused, c- 6-OHDA infused treated with Serotonin, d - 6-OHDA infused treated with GABA, e- 6-OHDA infused treated with BMC, f- 6-OHDA infused treated with Serotonin and BMC, g- 6-OHDA infused treated with GABA and BMC, h- 6-OHDA infused treated with Serotonin, GABA and BMC. There was an increased expression of NMDA2B receptors in the cerebral cortex of experimental rats when compared to control rats. The scale bars represent 75 μm.

Immunohistochemical analysis confirmed the receptor and gene expression data. mGLUR5 and NMDAR1 expression was significantly (P < 0.001) increased in the 6-OHDA infused rats compared to the control. Individual treatment of BMC didn't show any change. Combinational treatment significantly reversed the mean pixel value near to the control. (Figure 5, 6; Table 6)

**Table 6:** mGluR5 and NMDA2B expression in the Cerebral cortex of the experimental rats

Animal Status	Mean pixel value	
	
	mGluR5	NMDA2B
Control	23.25 ± 3.50	26 ± 3.1

6-OHDA	83.12 ± 3.23^a^	60 ± 6.1^a^

6-OHDA +5HT	67.12 ± 2.50^b, f^	50 ± 5.9^b,f^

6-OHDA +GABA	68.23 ± 3.05^b, f^	52 ± 5.7^b,f^

6-OHDA +BMC	79.33 ± 7.55^a^	59 ± 5.1^a^

6-OHDA +5HT + BMC	51.42 ± 5.93^c,e^	40 ± 5.2^c,e^

6-OHDA + GABA + BMC	53.77 ± 5.56^c,e^	42 ± 4.4^c,e^

6-OHDA +5HT + GABA+ BMC	35.69 ± 6.42^d^	30 ± 1.1^d^

Values are Mean ± S.E.M of 4-6 separate experiments. Each group consist 6-8 rats.

^a ^p < 0.001, ^b ^p < 0.01, ^c ^P < 0.05 when compared to Control,

^d ^p < 0.001, ^e ^p < 0.01, ^f ^P < 0.05 when compared to 6-OHDA group.

C - Control, 6-OHDA - 6-OHDA infused, 6-OHDA +5-HT - 6-OHDA infused treated with Serotonin, 6-OHDA +GABA - 6-OHDA infused treated with GABA, 6-OHDA +BMC- 6-OHDA infused treated with BMC, 6-OHDA +5-HT+BMC- 6-OHDA infused treated with Serotonin and BMC, 6-OHDA + GABA +BMC- 6-OHDA infused treated with GABA and BMC, 6-OHDA +5-HT + GABA+ BMC- 6-OHDA infused treated with Serotonin, GABA and BMC.

## Discussion

PD is classically characterized as a disorder resulting from the degeneration of dopaminergic neurons in the pars compacta of the substantia nigra. However, glutamatergic pathways play a leading role in the structural and functional organization of the cortico-basocortical loops involved in PD [27]. Changes in personality and moderate or mild cognitive debilitation are found in PD. Cerebral glucose metabolism is reduced in the cerebral cortex in PD patients suffering from cognitive impairment [28]. Metabolic and neuroimaging observations have recently documented decreased prefrontal and parietal 18F-fluorodeoxyglycose uptake in PD cases with mild cognitive deficits [29,30]. Recent observations have demonstrated complex I deficiency [31], and abnormal ATP synthase and inner protein membrane prohibitin expression levels [32] in the frontal cortex in PD. Several reports have highlighted the need of dopamine-glutamate coactivation for a number of cortical functions [33,34].

In the present study, we obtained decreased dopamine content in the cerebral cortex region which resulted in the increased expression of the glutamate activity. Increased glutamate content in the 6-OHDA infused rats leads to the up regulation of total glutamate and NMDA receptors. This was confirmed by the gene expression studies of mGluR5 and NMDA2B, where it showed an upregulation in 6-OHDA infused rats compared to control. The extracellular concentration of the glutamate in the CNS must be kept low to ensure a high signal to noise ratio during synaptic activation and to prevent excitotoxicity due to excessive activation of glutamate receptors [35]. Glutamate uptake into neurons and glial cells is important for the termination of glutamatergic transmission. They are essential for the maintenance of low extracellular levels of glutamate [36]. We observed a reduced expression of GLAST in 6-OHDA infused rats. The decreased glutamate transporter GLAST expression reduces the reuptake of the extracellular glutamate. Thus the results showed evidence for the dysfunction of the cerebral cortex that is a reflection for manifestation of abnormal behavioural patterns.

All of glutamate receptors couple positively to phospholipase C via guanine nucleotide binding proteins (G-proteins) whereby they stimulate phosphoinositide hydrolysis generating a second messenger cascade consisting of diacylglycerol and inositol 1,4,5 trisphosphate [37]. Jo *et al*., [38] demonstrated that NMDA and mGluR receptors mediate calcium release by stimulating IP3 and PKC. β1-adrenoceptors are highly expressed in PD which induced the up-regulation of cAMP/PKA signaling [39]. In our studies we observed an elevated cAMP and IP3 level in the cerebral cortex of 6-OHDA induced rats. The elevated IP3 level causes extra cellular release of Ca^2+^, which in turn enhanced metabolic stress on mitochondria that leads to excessive oxidative phosphorylation and increased production of reactive oxygen species. If the matrix Ca^2+ ^level rises too high, then deleterious changes in mitochondrial structure may occur. In particular, mitochondria can swell and rupture or undergo permeability transition, thereby releasing several pro-apoptotic factors into the cytoplasm, such as cytochrome C, second mitochondrial activator of caspases (SMAC/Diablo) or apoptosis-inducing factor (AIF) [40]. Our study showed an increased activity of bax gene expression in the cerebral cortex of the 6-OHDA infused rats which indicated the ROS mediated neurodegeneration in the cerebral cortex. Bax, one of the major pro-apoptotic family members, exerts its effects by compromising the membrane integrity leading to leakage of apoptogenic factors such as cytochrome c into the cytosol, resulting in caspase-3 activation and demise of the cell [41].

CREB is a transcription factor that plays an important role in neuronal survival, in part by controlling the transcription of neuroprotective genes [42]. The promoter regions of the genes for brain-derived neurotrophic factor (BDNF) and the pro-survival protein Bcl-2 contain cAMP response elements (CREs) [43]. 6-OHDA administration causes a decrease in transactivation of the CRE promotor, resulting in reduced expression of downstream CREB-regulated genes [44]. In the present study the gene expression of CREB was down regulated in cerebral cortex of 6-OHDA compared to control. Even though cAMP level was increased, the CREB expression was decreased. Enhanced activation of the glutamate receptors leads to the production of second messengers. But its acute and prolonged action triggers the cell death pathways by activating pro apoptotic genes like bax, bad and destabilizing jun- fos complex. The activation of apoptotic pathways down regulates the CREB expression thereby blocking the cAMP signaling cascade in PD rats. Down regulation of CREB is a consequence of apoptotic pathway activation and down regulation of muscarinic receptor function. These findings suggest that decreased CREB expression is the result of cell loss. BMC administration along with the 5-HT and GABA reduced the expression of apoptotic factors like bax so that CREB expression in these group reversed back to near control.

Normally an unstructured soluble protein, alpha-synuclein aggregates in the form of Lewy bodies and Lewy neurites in the frontal cortex in PD [32,45]. High concentrations of 6-OHDA results in neuronal death accompanied by a decrease of the monomeric form of alpha-synuclein, leading to both decreased synthesis of the protein and its increased mono-ubiquitination accompanied by nuclear translocation [46]. Studies by Pierson et al., [47] showed an increased level of unconjugated ubiquitin in the dorsal striatum of the dopamine depleted hemisphere. Normal alpha-synuclein expression is essential for the viability of primary neurons. Gene expression studies of alpha-synuclein in the cerebral cortex showed a significant down regulation in the 6-OHDA induced rats compared to control. This indicates the reduced expression of normal alpha-synuclein in the PD rats. Up regulation of ubiquitin carboxy-terminal hydrolase gene expression in cerebral cortex confirmed the increased level of unconjugated ubiquitin in the 6-OHDA induced rats. Combinational treatment significantly reversed these changes back to control.

BMC, the non-hematopoietic precursor cells (i.e. mesenchymal stem and progenitor cells) in bone marrow, offer an alternative source of cells for treatment of neurodegenerative diseases and central nervous system (CNS) injury. These cells normally differentiate into bone, cartilage and adipose tissue [48], but can be induced to differentiate into cells with surface markers characteristic of neurons [49,50]. Autologous BMC to treat neurological disorders offers several unique advantages over other cell replacement therapies. For one, immunological reactions are avoided and it also bypasses many of the ethical issues that surround the use of embryonic cells. Recent study shows that post-symptomatic treatment with granulocyte colony-stimulating factor (G-CSF) in 1-methyl-4-phenyl-1,2,3, 6-tetrahydropyridine (MPTP) mouse model of PD rats can promote the regeneration of dopaminergic neurons in the SNpc and restore nigrostriatal function [51]. 5-HT and GABA are involved in a variety of cellular processes which includes neurogenesis, proliferation and morphology [9-15]. Our study demonstrated that BMC administration alone cannot reverse the above said molecular changes occurring during PD. We found that 5-HT, GABA and BMC in combination potentiates a restorative effect by reversing the alterations in glutamate receptor binding and gene expression that occur during Parkinson's disease. Thus, it is evident that 5-HT and GABA along with BMC to 6-OHDA infused rats renders protection against oxidative, related motor and cognitive deficits which makes them clinically significant for cell-based therapy.

## Abbreviations

PD: Parkinson's disease; BMC: Bone marrow cells; GABA: Gamma aminobutyric acid; 5-HT: Serotonin; CREB: Cyclic AMP response element binding protein.

## Competing interests

The authors declare that they have no competing interests.

## Authors' contributions

NMS and CSP designed research. NMS, JP, KPK, AM, and CR carried out the experiments and drafted manuscript. All authors read and approved the final manuscript.
